# Optimized flow compensation for contrast-enhanced T1-weighted Wave-CAIPI 3D MPRAGE imaging of the brain

**DOI:** 10.1186/s41747-023-00351-y

**Published:** 2023-07-03

**Authors:** Azadeh Tabari, Min Lang, Komal Awan, Wei Liu, Bryan Clifford, Wei-Ching Lo, Daniel Nicolas Splitthoff, Stephen Cauley, Otto Rapalino, Pamela Schaefer, Susie Y. Huang, John Conklin

**Affiliations:** 1 Department of Radiology, Athinoula A. Martinos Center for Biomedical Imaging, Massachusetts General Hospital, 55 Fruit Street, Charlestown, Boston, MA 02114 USA; 2 Harvard Medical School, Boston, MA USA; 3 Siemens Shenzhen Magnetic Resonance Ltd., Shenzhen, China; 4 Siemens Healthcare GmbH, Boston, MA USA; 5 Siemens Medical Solutions, Erlangen, Germany; 6 Harvard-MIT Health Sciences and Technology, Massachusetts Institute of Technology, Cambridge, MA USA

**Keywords:** Artifacts, Magnetic resonance imaging, Phantoms (imaging), Pulsatile flow

## Abstract

Flow-related artifacts have been observed in highly accelerated T1-weighted contrast-enhanced wave-controlled aliasing in parallel imaging (CAIPI) magnetization-prepared rapid gradient-echo (MPRAGE) imaging and can lead to diagnostic uncertainty. We developed an optimized flow-mitigated Wave-CAIPI MPRAGE acquisition protocol to reduce these artifacts through testing in a custom-built flow phantom. In the phantom experiment, maximal flow artifact reduction was achieved with the combination of flow compensation gradients and radial reordered *k*-space acquisition and was included in the optimized sequence. Clinical evaluation of the optimized MPRAGE sequence was performed in 64 adult patients, who all underwent contrast-enhanced Wave-CAIPI MPRAGE imaging without flow-compensation and with optimized flow-compensation parameters. All images were evaluated for the presence of flow-related artifacts, signal-to-noise ratio (SNR), gray-white matter contrast, enhancing lesion contrast, and image sharpness on a 3-point Likert scale. In the 64 cases, the optimized flow mitigation protocol reduced flow-related artifacts in 89% and 94% of the cases for raters 1 and 2, respectively. SNR, gray-white matter contrast, enhancing lesion contrast, and image sharpness were rated as equivalent for standard and flow-mitigated Wave-CAIPI MPRAGE in all subjects. The optimized flow mitigation protocol successfully reduced the presence of flow-related artifacts in the majority of cases.

**Relevance statement**

As accelerated MRI using novel encoding schemes become increasingly adopted in clinical practice, our work highlights the need to recognize and develop strategies to minimize the presence of unexpected artifacts and reduction in image quality as potential compromises to achieving short scan times.

**Key points**

• Flow-mitigation technique led to an 89–94% decrease in flow-related artifacts.

• Image quality, signal-to-noise ratio, enhancing lesion conspicuity, and image sharpness were preserved with the flow mitigation technique.

• Flow mitigation reduced diagnostic uncertainty in cases where flow-related artifacts mimicked enhancing lesions.

**Graphical Abstract:**

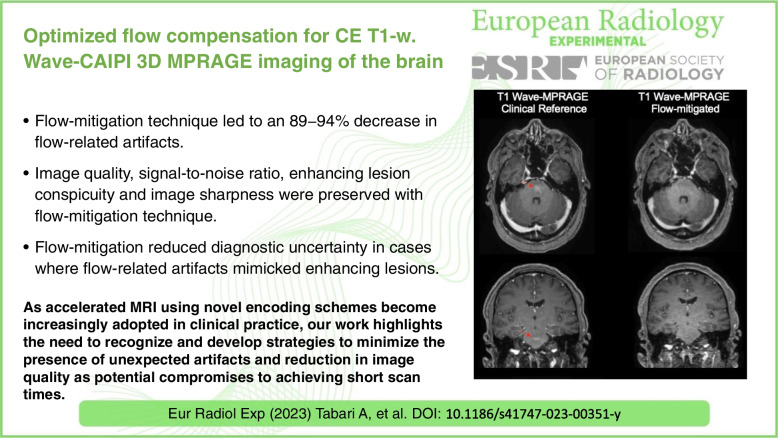

## Background

In recent years, accelerated magnetic resonance imaging (MRI) acquisition techniques have been increasingly adopted to meet the growing demand for medical imaging [1–4]. Acceleration techniques are continuously being refined to balance image quality, artifact, and scan time [5–8]. Wave-controlled aliasing in parallel imaging (Wave-CAIPI) is a relatively new parallel acquisition technique developed in the last decade that combines two-dimensional-CAIPI shifts and bunch phase encoding to maximize three-dimensional (3D) coil sensitivity and achieve controlled aliasing in all three spatial directions [5, 6], resulting in high acceleration factors with negligible g-factor penalty. Wave-CAIPI has been validated for clinical 3D volumetric T1-weighted magnetization-prepared rapid gradient echo (MPRAGE), fluid-attenuated inversion recovery, and susceptibility-weighted sequences, showing equivalent visualization of pathology and overall diagnostic quality compared to their conventional counterparts [9–14].

Flow artifacts are caused by pulsatile laminar flow that produces a complex multilayered band from flow-related dephasing and can propagate in the phase encoding direction. These flow-related artifacts are well known in MRI and may have anomalous appearances, depending on the *k*-space sampling pattern. Flow-related artifacts have been observed in contrast-enhanced Wave-CAIPI 3D T1-weighted MPRAGE and have an atypical appearance, manifesting as smearing of T1 hyperintense signal in the brainstem, subcortical nuclei, and other areas of the brain parenchyma [11]. Such artifacts introduce a diagnostic conundrum for the interpreting radiologist as they can mimic enhancing lesions and may require callback for repeat imaging with conventional non-accelerated MR sequences, posing a critical barrier to wider clinical adoption of this technique.

The goal of this study was to characterize the source of flow-related artifacts in contrast-enhanced Wave-CAIPI 3D T1-weighted MPRAGE using a novel flow phantom and develop an optimized flow-mitigated acquisition. The optimized protocol was then deployed in a clinical setting, and the resulting image quality and diagnostic performance were evaluated against the same sequence performed without flow mitigation.

## Methods

### Flow phantom experiment

**Fig. 1:**
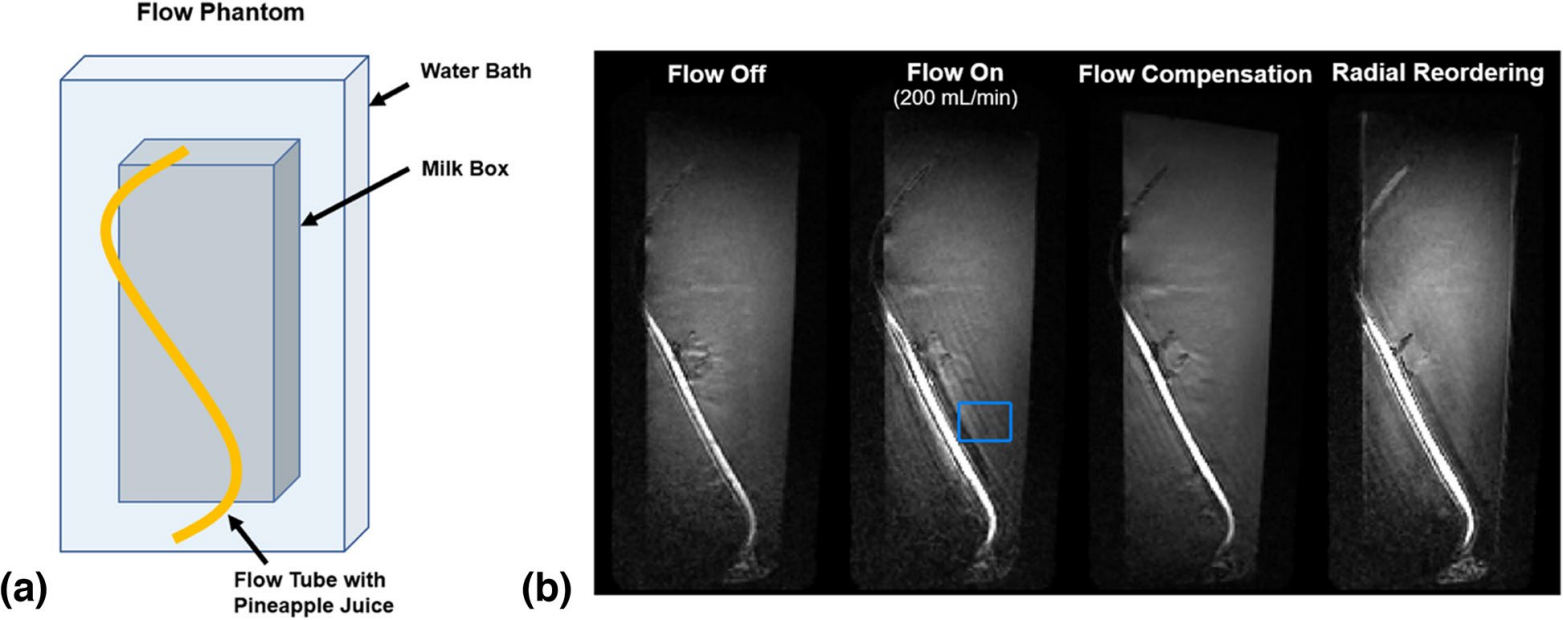
Schematic diagram (**a**) and representative images (**b**) of experiments in a flow phantom consisting of pineapple juice in tubing driven by a peristaltic pump. Wave magnetization-prepared rapid gradient-echo (MPRAGE) images with flow on demonstrate pronounced flow-related artifact, which is improved with flow compensation and the use of radial reordering of *k*-space as described in the proposed flow mitigation protocol (acquisition parameters in Table 1). The coefficients of variation were computed for each image in the region of interest depicted by the blue square in the flow on image. They were 0.1690 without flow, 0.2931 with flow on, 0.1262 with flow on and flow compensation gradients, and 0.2020 with flow on and radial reordering

A flow phantom was constructed consisting of a water bath containing tubing filled with pineapple juice (Fig. 1a). Pineapple juice can be used as an MRI contrast agent due to its short T1 relaxation time and was used to mimic post-contrast imaging in the phantom [15]. Flow was controlled in the tubing by a peristaltic pump (4-mm diameter, Runze Fluid, Nanjing City, China) and used to simulate vascular flow (~ 200 mL/min) in the cerebral vasculature.

Flow mitigation strategies were tested using our flow phantom: (a) flow compensation gradients based on first-order gradient moment nulling (GMN) and (b) radial reordering of *k*-space acquisition. The level of flow artifact reduction was quantified by computing the coefficient of variation in a region of interest (ROI).

### Conventional and flow-mitigated Wave-MPRAGE protocols

Flow compensation techniques were incorporated into the Wave-MPRAGE protocol and tested iteratively to determine the optimal parameters for mitigating flow-related artifacts. The flow compensation strategies implemented were the addition of flow compensation (*i.e.*, GMN) gradients and the use of a radial reordering of the *k*-space data acquisition. Gradient moment nulling is the process of modifying a gradient waveform in order to make a pulse sequence more immune to image artifacts arising from motion (*i.e.*, blood flow) [16–18]. GMN can reduce signal loss and ghosting image artifacts that occur when there is pulsatile flow or periodic motion by preventing view-to-view phase fluctuations due to physiological motion and thereby preventing motion artifacts and intensity loss, but higher order moments need to be accounted for [19]. We used first-order GMN in the proposed flow compensation scheme to compensate for flow with constant velocity. The combination of flow compensation gradients with radial reordering used in our final optimized protocol helped mitigate sensitivity to pulsatile flow. Radial reordering of *k*-space acquisitions is well known to facilitate motion-robust imaging because the high signal intensity echoes near the center of *k*-space are acquired much closer together in time, making them as consistent as possible. Importantly, the increased robustness to motion provided by radial reordering is not limited to constant flow [20, 21].

In addition to first-order GMN and radial reordering (available as on/off options in the protocol parameters), the readout bandwidth was increased from 200 to 360 Hz/pixel, the number of wave cycles was reduced from 16 to 9, and elliptical scanning (sampling) was used. These additional changes were all necessitated by the use of GMN and radial *k*-space reordering. For a fixed bandwidth, the addition of GMN increases the minimum allowable echo time (at a bandwidth of 200 Hz/pixel, the minimum echo time increased from 3.47 to 5.99 ms). Increasing the bandwidth reduced the minimum echo time to 4.26 ms and preserved tissue contrast. As a result of increasing the bandwidth, the research application automatically reduced the number of wave cycles from 16 to 9 to maintain the wave frequency. Lastly, the use of elliptical scanning kept the acquisition time of the two protocols nearly matched despite the slight increase in TR required for radial reordering (2.33 min for conventional Wave MPRAGE *versus* 2.28 min for the flow-mitigated Wave-MPRAGE protocol). The detailed imaging parameters of the original and flow-mitigated Wave-MPRAGE protocols are summarized in Table 1. The contrast administration protocol was as follows: gadoterate meglumine (Liebel-Flarsheim Company LLC, Raleigh, NC, USA), 0.2 mL/1 kg of body weight, slow hand injection with 10 mL saline bolus.

**Table 1:** Acquisition parameters for the conventional Wave-CAIPI 3D T1-MPRAGE and flow-mitigated Wave-CAIPI 3D T1-MPRAGE protocols

Parameter	Conventional Wave-MPRAGE	Flow-mitigated Wave-MPRAGE
Resolution (mm^3^)	1 × 1 × 1	1 × 1 × 1
Acceleration	4	4
Turbo factor	192	192
Acquisition time (min)	2.33	2.28
TE/TI/TR (ms)	3.47/900/2000	4.26/900/2440
Field of view (mm)	256 × 256	256 × 256
Bandwidth (Hz/pixel)	200	360
Wave cycle number	16	9
Reordering	Linear	Radial
Elliptical scanning	No	Yes
Flow compensation	Off	On

*3D* Three-dimensional, *CAIPI* Controlled aliasing in parallel imaging, *MPRAGE* Magnetization-prepared rapid acquisition gradient-echo

All clinical exams included both conventional Wave-CAIPI post-contrast 3D T1 MPRAGE sequences based on standardized institutional brain MRI protocols followed by the optimized flow-mitigated version of Wave-CAIPI post-contrast 3D T1 MPRAGE sequence. Images were obtained using a 20-channel head and neck receiver coil array.

### Patient selection

This single-institution pilot research study was approved by the local Human Research Committee of the Institutional Review Board. The study was compliant with the Health Insurance Portability and Accountability Act (HIPAA). We enrolled adult patients who were scheduled for outpatient contrast-enhanced brain MRI exams on a 3-T MRI system (MAGNETOM Vida, Siemens Healthcare, Erlangen, Germany) during October and November 2022. The exclusion criteria were the same as those for routine clinical MR imaging. Written informed consent was not required since no significant time (~ 2 min) was added to each exam.

### Image analysis

For all clinical studies, two neuroradiologists (S.H. and J.C., with 12 and 10 years of experience, respectively) independently evaluated the anonymized image datasets, including conventional and flow-mitigated Wave-CAIPI MPRAGE images acquired in the sagittal plane and reformatted in all three planes on an independent workstation. Readers were blinded to the imaging sequence and clinical diagnosis. To emulate image review in a realistic clinical setting, the readers were allowed to adjust the window-level settings for each sequence according to their own preferences. The images were randomly assigned labels of A or B and were opened side by side on a single monitor. The raters compared the two sequences according to the following characteristics: the presence of flow artifacts, signal-to-noise ratio (SNR), gray-white matter contrast, enhancing lesion contrast, and image sharpness. Images were graded using a 3-point Likert scale. Grade 1 indicated image A was preferred to image B, grade 0 indicated no significant difference between the two image series, and grade -1 indicated image B was preferred to image A.

### Quantitative evaluation

The contrast-to-noise ratio (CNR) and SNR measurements were performed for conventional and flow-mitigated Wave-CAIPI MPRAGE images. The ROIs were placed on the left basal ganglia (gray matter) and on the left inferior frontal subcortical white matter to measure signal intensity. For each subject, noise was sampled using 30-voxel ROIs in air-containing regions above the left aspect of the head. The SD of the background noise was calculated for the same ROIs on both image series. We calculated the CNR by dividing the difference into gray and white matter intensities by the SD of the background noise. The SNR in gray and white matter was calculated by dividing the mean signal intensity by the SD of the background noise.

### Statistical analysis

The presence of flow artifacts, SNR, gray-white matter contrast, enhancing lesion contrast, and image sharpness were compared between the two groups using the Wilcoxon rank sum test. Statistical calculations were performed using RStudio (RStudio, Boston, MA, USA). *p*-values < 0.05 were deemed statistically significant.

## Results

In the flow phantom experiments, the combination of flow compensation gradients and radial reordering *k*-space acquisition provided the greatest level of flow artifact reduction. The results of the phantom experiments are provided in Fig. 1b. The estimated coefficients of variation in the ROI were 0.1690 without flow, 0.2931 with flow on, 0.1262 with flow on and flow compensation gradients, and 0.2020 with flow on and radial reordering.

A total of 64 patients (41 females, 64%; mean age 56 years, age range 29–85 years) met the inclusion criteria and were enrolled consecutively in the clinical evaluation study. Contrast-enhanced brain MRI including the conventional Wave-CAIPI MPRAGE sequence followed by the flow-mitigated version of Wave-MPRAGE was successfully acquired in all 64 cases. The study patients were imaged for the following clinical indications: initial work-up or follow-up of brain tumor (*n* = 34; 53% of all patients), headache (*n* = 12; 19%), visual loss (*n* = 3; 5%), meningioma (*n* = 2; 3%), subdural hemorrhage (*n* = 2; 3%), mental status change (*n* = 2; 3%), and others (*n* = 9; 14%). In the clinical evaluation study, the optimized flow mitigation protocol was judged to have successfully reduced flow-related artifacts in 57 of 64 (89%) cases by rater 1 and 60 of 64 (94%) cases by rater 2. For subjective evaluations, SNR, gray-white matter contrast, enhancing lesion contrast, and image sharpness were rated by both raters as equivalent to the conventional and flow-mitigated Wave-MPRAGE images in all patients (Table 2). The optimized flow mitigation protocol was the preferred sequence for reduced flow-related artifacts by both raters (*p* = 0.001).

**Table 2:** Summary of subjective assessments by rater 1 and rater 2 (R1 and R2)

	**Signal-to-noise ratio**		**Sharpness**		**Gray-white matter contrast**		**Enhancing lesion contrast**		**Pulsation artifact**	
	R1	R2	R1	R2	R1	R2	R1	R2	R1	R2
**Conventional Wave-CAIPI preferred**	–	–	–	–	–	–	–	–	5% (3/64)	−
**Equivalent**	100% (64/64)	100% (64/64)	100% (64/64)	100% (64/64)	100% (64/64)	100% (64/64)	100% (64/64)	100% (64/64)	6% (4/64)	6% (4/64)
**Flow-mitigated Wave-CAIPI preferred**	–	–	–	–	–	–	–	–	89% (57/64)	94% (60/64)

**Fig. 2:**
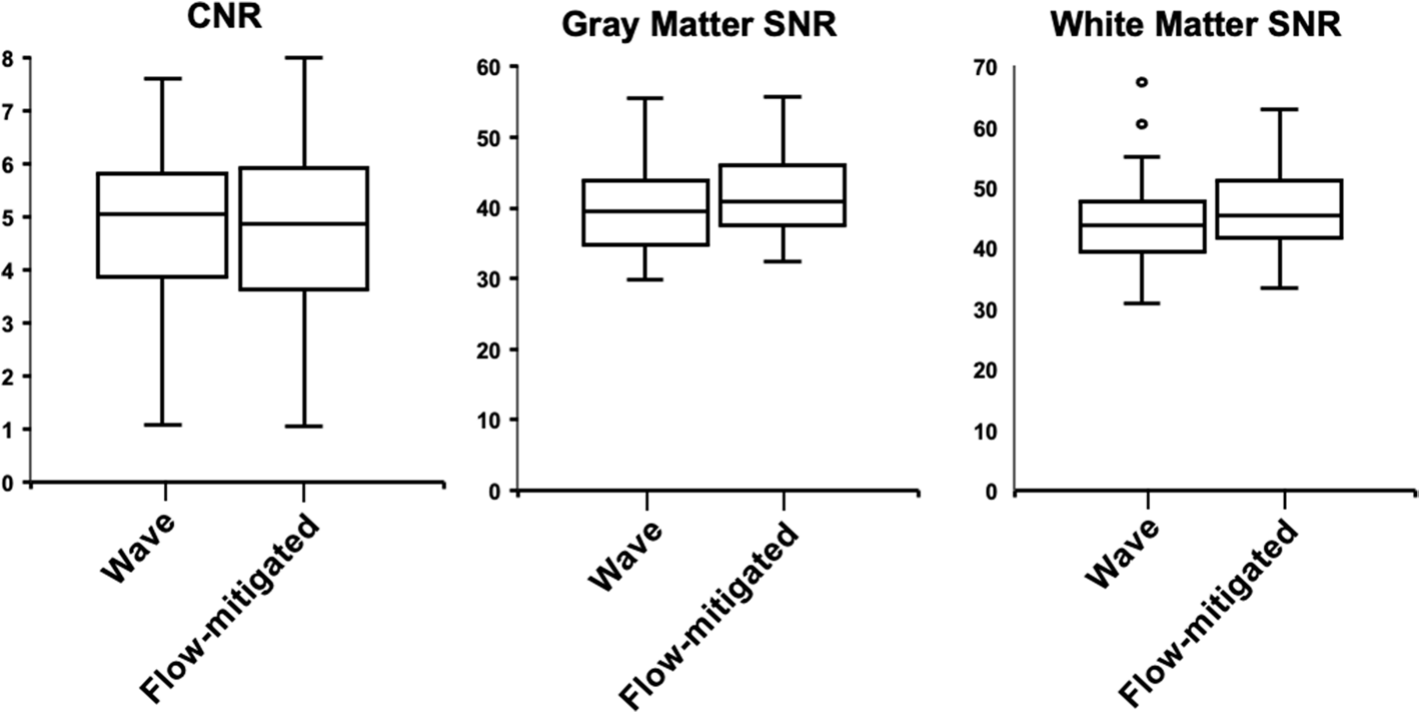
Boxplot charts demonstrating the distribution of contrast-to-noise ratio (CNR) and signal-to-noise ratio (SNR) in the gray matter and white matter in conventional Wave-MPRAGE (magnetization-prepared rapid gradient-echo) and flow-mitigated MPRAGE images. CNRs, as well as gray matter and white matter SNRs, were comparable in the Wave-MPRAGE images and flow-mitigated MPRAGE images (*p* = 0.612, *p* = 0.066, and *p* = 0.087, respectively)

Quantitative assessment of SNR and CNR showed no significant difference for the mean CNR as well as SNR in the gray and white matter (*p* = 0.612, *p* = 0.066, and *p* = 0.087, respectively) between conventional Wave- and flow-mitigated MPRAGE images (Fig. 2).

**Fig. 3:**
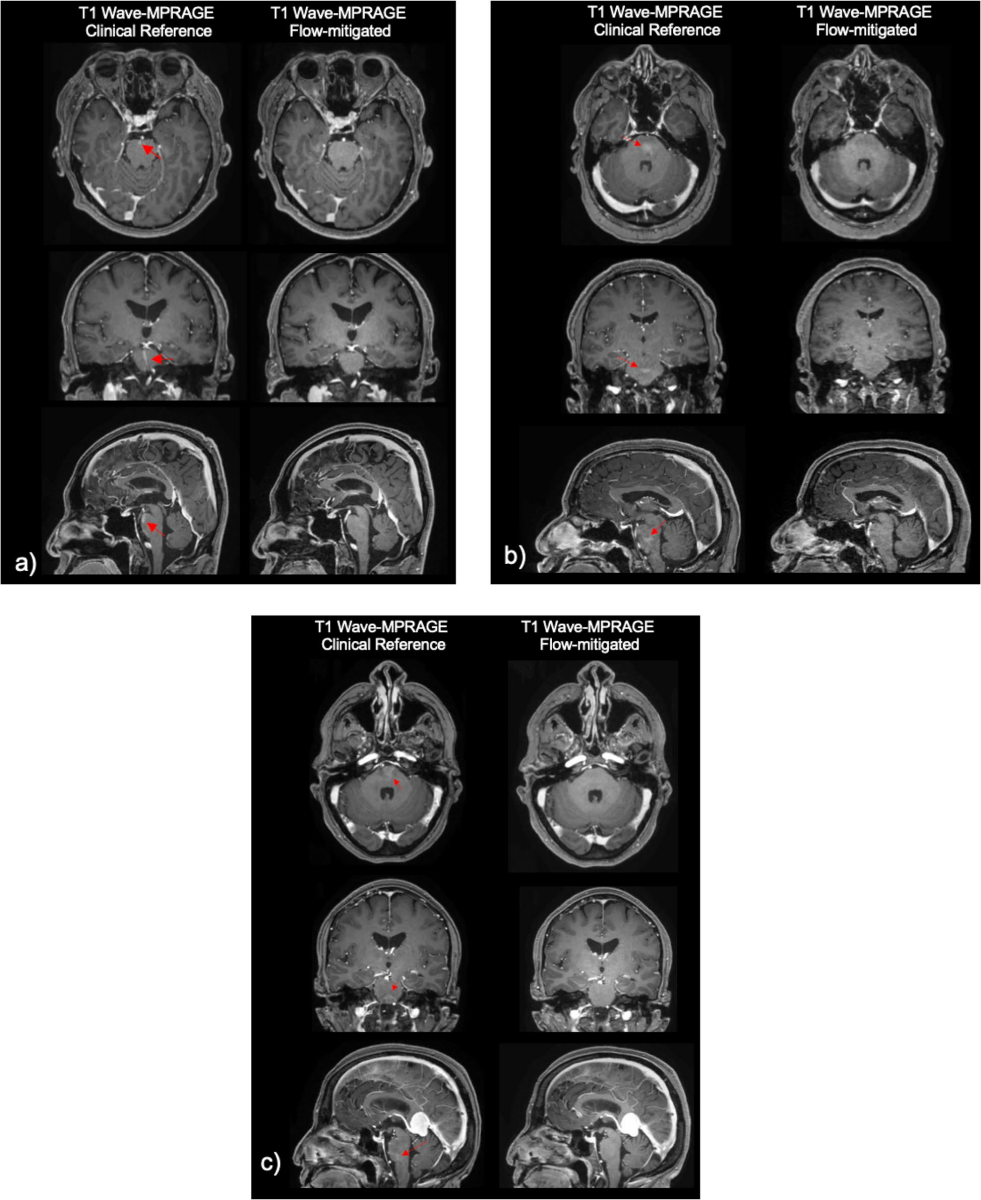
Representative examples of flow-related artifacts on conventional wave-controlled aliasing in parallel imaging (CAIPI) post-contrast T1-weighted magnetization-prepared rapid gradient-echo (MPRAGE) images that were reduced or eliminated on the flow-mitigated same sequence. Arrows point to the enhancing pseudo-lesions visible on multiplanar reformats of the conventional images. The abnormal enhancement was artifactual and corrected on the flow-mitigated Wave-MPRAGE images. Patient 1 (**a**): vascular pseudo-enhancement due to flow-related artifact in the ventromedian pons. The pseudo-enhancement is not seen on the flow-mitigated images. Patient 2 (**b**): an enhancing pseudo-lesion in the inferior pons. The flow-related pseudo-enhancement is not seen on the flow-mitigated images. Patient 3 (**c**): area of apparent enhancement on the conventional Wave-MPRAGE images mimics the appearance of an enhancing lesion in the pons, likely arising from flow in the basilar artery/venous plexus. No abnormal enhancement was seen in the pons on the flow-mitigated images

**Fig. 4:**
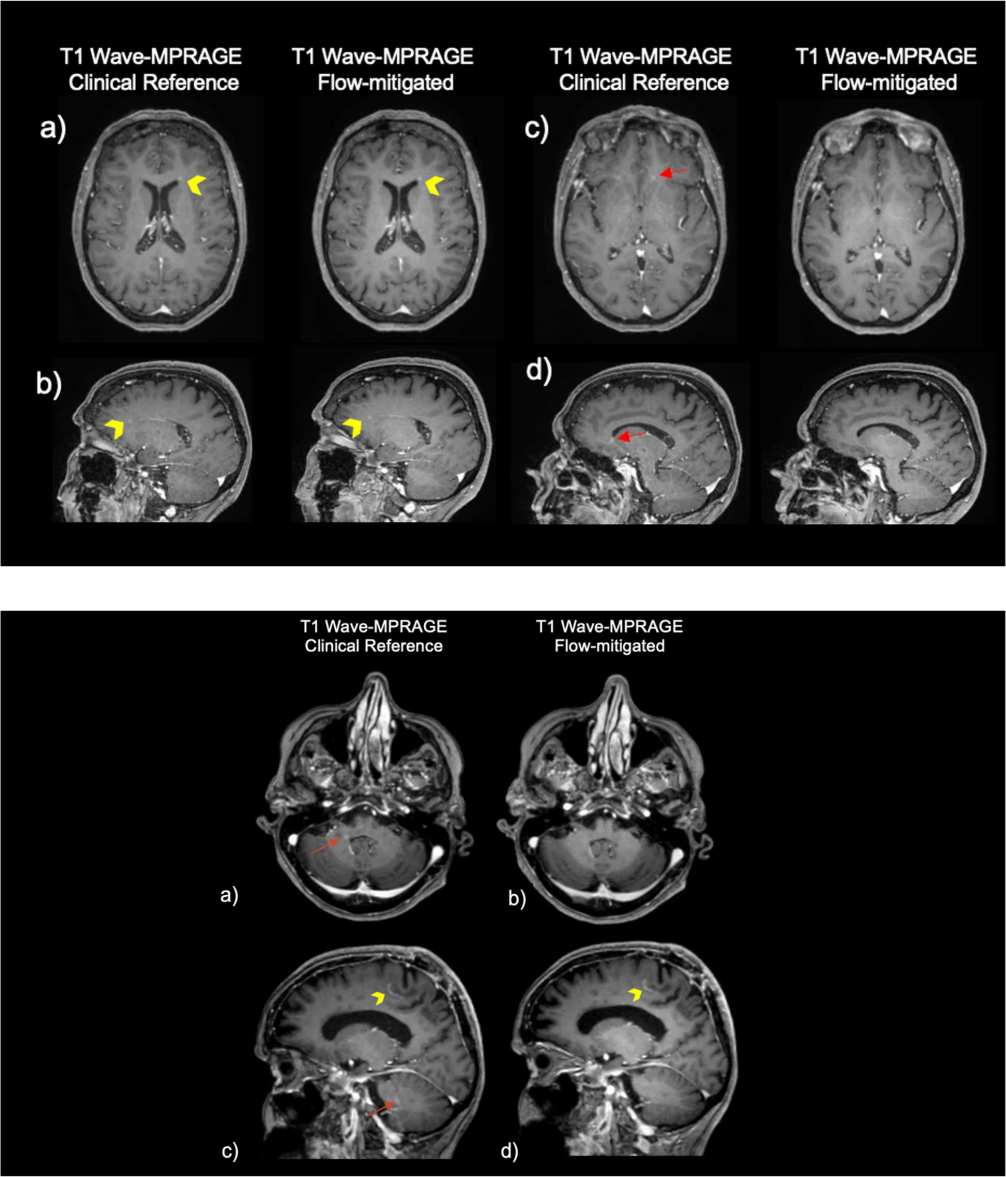
Two representative pathologic cases with true enhancing lesions as well as flow-related artifacts mimicking additional enhancing lesions. The flow-mitigated technique was able to discern true enhancing lesions from the flow-related artifact, which were absent on flow-mitigated post-contrast T1-weighted MPRAGE (magnetization-prepared rapid gradient-echo) images. Flow mitigation improved diagnostic accuracy in both cases and more accurately assessed the degree of disease burden. Patient 1 (P1): 71-year-old female with stage 4 breast cancer who underwent contrast-enhanced brain MRI to screen for intracranial metastatic disease. Axial (**a**) and sagittal (**b**) images show a 3-mm enhancing focus in the left forceps minor (arrowhead) on the conventional and flow-mitigated Wave-MPRAGE images, consistent with a metastatic lesion, demonstrating true *versus* false enhancing focuses on T1-weighted Wave-MPRAGE images. Axial (**c**) and sagittal (**d**) views show a punctate focus of apparent enhancement in the left inferior frontal lobe on the conventional Wave-MPRAGE images that is not present on the corresponding flow-mitigated Wave-MPRAGE images, suggesting the area of enhancement was artifactual. Patient 2 (P2): 82-year-old female with progressive multifocal leukoencephalopathy showing a small focus of apparent enhancement (arrows) on axial (**a**) and sagittal (**c**) views of conventional T1-weighted post-contrast Wave-MPRAGE imaging. No enhancement was noted on the corresponding axial (**b**) and (**d**) sagittal flow-mitigated Wave-MPRAGE images, suggesting that the area of apparent enhancement was consistent with flow-related artifact. Curvilinear enhancement is also seen in the posterior frontal lobe subcortical U-fiber (arrowhead), corresponding to a white matter demyelinating lesion associated with the patient’s known diagnosis of progressive multifocal leukoencephalopathy

Figure 3 shows the representative flow-related artifacts in the pons arising from the basilar artery and venous plexus on conventional Wave-MPRAGE (without flow-compensation) that were subsequently reduced or eliminated on the optimized flow-mitigated Wave-MPRAGE images. Figure 4a highlights several foci of apparent enhancement in the left frontal lobe in a patient with stage 4 breast cancer undergoing contrast-enhanced brain MRI for evaluation of intracranial metastatic disease. The flow-mitigated Wave-MPRAGE images helped reduce diagnostic uncertainty by revealing the lesions that were most likely due to flow-related artifacts and did not represent true enhancing lesions. Figure 4b shows a case with progressive multifocal leukoencephalopathy demonstrating a small focus of apparent enhancement in the right cerebellar peduncle on the conventional T1-weighted post-contrast Wave-MPRAGE imaging. No enhancement was observed on the flow-mitigated Wave-MPRAGE images, suggesting that the area of apparent enhancement was due to flow-related artifact and not related to the underlying progressive multifocal leukoencephalopathy process.

## Discussion

This study evaluated an optimized flow-mitigated Wave-MPRAGE protocol for reducing flow-related artifacts in post-contrast T1-weighted imaging. Flow mitigation strategies were first tested and optimized in a flow phantom and were subsequently deployed and validated clinically in a cohort of 64 patients undergoing contrast-enhanced brain MRI in an outpatient setting. Flow mitigation was successful at reducing flow-related artifacts in most cases without sacrificing SNR, gray-white matter contrast, enhancing lesion conspicuity, or image sharpness.

As fast MRI utilizing novel spatial encoding schemes like Wave-CAIPI become increasingly adopted in clinical practice, this study highlights the need to minimize the presence of unexpected artifacts and reduction in image quality as potential compromises to achieving short scan times. Flow-related artifacts due to hyperdynamic flow in the cerebrospinal fluid or vasculature may be particularly pronounced in certain populations, *e.g.*, in children, and may manifest in an unfamiliar way, demonstrated in the presented figures as streaking and smearing of bright signal that occurs to varying degrees in post-contrast Wave-CAIPI MPRAGE images without flow compensation [22]. The proposed flow mitigation strategies described here may thus be beneficial to integrate into other Wave-CAIPI contrasts to improve image quality.

Artifacts in MRI may be confused with pathology and may reduce the quality of examinations [23]. Attempts have been made to classify MRI artifacts based on their location, source, and appearance [24]. However, even well-known artifacts can present in unexpected manners when new encoding methods are used [25]. Flow artifacts caused by pulsatile laminar flow pose a challenge to differentiating true pathology from artifacts [23, 26]. In our work, we have applied MR physics concepts to shed light on the causes of these artifacts and develop techniques to minimize their confounding effect on the resulting images.

In this study, flow-mitigated Wave-MPRAGE was the preferred diagnostic sequence for both neuroradiologists due to decreased flow-related artifacts. The results suggest that flow-mitigated T1-weighted post-contrast Wave-MPRAGE was equivalent to conventional T1-weighted post-contrast Wave-MPRAGE for most indications when evaluating SNR and other qualitative image metrics such as gray-white matter contrast, enhancing lesion contrast and image sharpness. A significant decrease in flow-rated artifacts was achieved when using an optimized flow mitigation protocol compared to the conventional Wave-MPRAGE protocol without flow mitigation.

This study has several limitations. For the phantom experiments, the pump did not support the generation of pulsatile flow, and as a result, the added robustness to pulsation-related artifacts provided by radial reordering is not obvious in these results. For the clinical study, the small sample size (*n* = 64) may limit the generalizability of the findings. Furthermore, the mean SNR in gray and white matter was higher in flow-mitigated Wave-MPRAGE and close to reaching statistical significance (0.066 and 0.087, respectively) but may have been underpowered due to the sample size. Nevertheless, a wide range of enhancing pathologies were visualized, and the number of patients with enhancing lesions was adequate to demonstrate a significant improvement in image quality with flow mitigation. Another potential limitation was the fixed order of acquisition: Wave-MPRAGE images without flow compensation were always acquired first, followed by the Wave-MPRAGE images with flow compensation. The conspicuity of enhancing lesions increases with time elapsed from contrast administration, which would increase the detectability of enhancing lesions in the sequence acquired last but would also make any flow artifacts related to contrast enhancement more obvious. Thus, we considered the clear improvement in image quality with flow mitigation as sufficient to overcome any potential bias incurred by the fixed order of post-contrast image acquisition.

In conclusion, the flow-mitigated Wave-MPRAGE sequence significantly reduced flow-related artifacts without sacrificing the overall image quality. As accelerated MRI using novel encoding schemes become increasingly adopted in clinical practice, our work highlights the need to recognize and develop strategies to minimize the presence of unexpected artifacts and reduction in image quality as potential compromises to achieving short scan times.

## Data Availability

The datasets used and/or analyzed during the current study are available from the corresponding author upon reasonable request.

## Abbreviations

3D: Three-dimensional
CAIPI: Controlled aliasing in parallel imaging
CNR: Contrast-to-noise ratio
GMN: Gradient moment nulling
MPRAGE: Magnetization-prepared rapid gradient-echo
MRI: Magnetic resonance imaging
SNR: Signal-to-noise ratio
